# Advances in genetic technologies result in improved diagnosis of mismatch repair deficiency in colorectal and endometrial cancers

**DOI:** 10.1136/jmedgenet-2020-107542

**Published:** 2021-01-15

**Authors:** D Gareth Evans, Fiona Lalloo, Neil AJ Ryan, Naomi Bowers, Kate Green, Emma R Woodward, Tara Clancy, James Bolton, Rhona J McVey, Andrew J Wallace, Katy Newton, James Hill, Raymond McMahon, Emma J Crosbie

**Affiliations:** 1 Division of Evolution and Genomic Medicine, The University of Manchester, Manchester, UK; 2 Clinical Genetics Service, Manchester Centre for Genomic Medicine, North-West Genomics Laboratory Hub, Manchester University NHS Foundation Trust, Manchester, Greater Manchester, UK; 3 Division of Cancer Sciences, The University of Manchester, Manchester, UK; 4 Department of Obstetrics and Gynaecology, Manchester University NHS Foundation Trust, Manchester, Greater Manchester, UK; 5 Department of Pathology, Manchester University NHS Foundation Trust, Manchester, Greater Manchester, UK; 6 Department of Surgery, Manchester University NHS Foundation Trust, Manchester, Greater Manchester, UK

**Keywords:** genetic predisposition to disease, genetic testing, surgical oncology

## Abstract

**Background:**

Testing cancers for mismatch repair deficiency (dMMR) by immunohistochemistry (IHC) is a quick and inexpensive means of triaging individuals for germline Lynch syndrome testing. The aim of this study was to evaluate tumour dMMR and the prevalence of Lynch syndrome in patients referred to the Manchester Centre for Genomic Medicine, which serves a population of 5.6 million.

**Methods:**

Tumour testing used IHC for MMR proteins with targeted *BRAF* and *MLH1* promotor methylation testing followed by germline mutation and somatic testing as appropriate.

**Results:**

In total, 3694 index tumours were tested by IHC (2204 colorectal cancers (CRCs), 739 endometrial cancers (ECs) and 761 other), of which 672/3694 (18.2%) had protein loss, including 348 (9.4%) with MLH1 loss. MLH1 loss was significantly higher for 739 ECs (15%) vs 2204 CRCs (10%) (p=0.0003) and was explained entirely by higher rates of somatic *MLH1* promoter hypermethylation (87% vs 41%, p<0.0001). Overall, 65/134 (48.5%) patients with MLH1 loss and no *MLH1* hypermethylation or *BRAF* c.1799T>A had constitutional *MLH1* pathogenic variants. Of 456 patients with tumours showing loss of MSH2/MSH6, 216 (47.3%) had germline pathogenic variants in either gene. Isolated PMS2 loss was most suggestive of a germline MMR variant in 19/26 (73%). Of those with no germline pathogenic variant, somatic testing identified likely causal variants in 34/48 (71%) with MLH1 loss and in *MSH2/MSH6* in 40/47 (85%) with MSH2/MSH6 loss.

**Conclusions:**

Reflex testing of EC/CRC leads to uncertain diagnoses in many individuals with dMMR following IHC but without germline pathogenic variants or *MLH1* hypermethylation. Tumour mutation testing is effective at decreasing this by identifying somatic dMMR in >75% of cases.

## Introduction

Colorectal cancer (CRC) and endometrial cancer (EC) are two of the most common malignancies in humans. They are both characterised by having a relatively high rate of mismatch repair deficiency (dMMR) and similar germline rates (3%) of pathogenic variants in MMR genes.^1^ CRC is the third most common cancer in men and women.^2^ EC is the most common gynaecological cancer in high-income countries, and its incidence is rising rapidly.^3^ Although environmental causes such as diet (CRC) and obesity (particularly EC)^4^ and decreased parity (EC) are major contributors to incidence, a significant minority of both cancers (3%) are caused by Lynch syndrome (LS).^1 5^ LS is an inherited susceptibility to malignancies associated with dMMR. Around 1 in 280 of the general population is heterozygous for a pathogenic variant in an MMR gene, *MLH1*, *MSH2* (including *deletions of EPCAM*), *MSH6* or *PMS2* (path_MMR), the vast majority of whom are undiagnosed.^5–8^ Path_MMR heterozygotes have an averaged risk to age 70 years of EC, CRC and ovarian cancer (OC) of 35%, 46% and 11%, respectively,^9^ although these vary by gene with lower risks of *PMS2*. These likelihoods are substantially higher than those of the general population for EC (3%), CRC (4%) and OC (1%).^10^

Since the discovery of the MMR genes in 1993–1994, germline testing has been targeted towards those most likely to have an inherited pathogenic variant. The Amsterdam criteria were developed in 1991,^11^ primarily to select high-risk families, therefore requiring a substantial family history of CRC. While 45%–60% of index cases in families fulfilling criteria are path_MMR variant carriers,^12^ the criteria have low sensitivity.^13 14^ The addition of other characteristic tumours of LS, such as EC, OC and urothelial cancers,^15^ add little to either the detection rate^12^ or sensitivity.^13 14^ The less restrictive Bethesda guidelines were developed in 1997,^16^ which improved sensitivity but resulted in many more samples being tested without detection of all path_MMR variants.^13 14 17 18^ More recently, the concept of universal testing of CRC has gained ground^18–22^ and is now recommended national guidance in a number of countries for CRC.^20 21^ This is also gaining traction for EC^23^ and is now recommended by the National Institute for Health and Care Excellence in the UK.^24^

We have evaluated our prescreening strategy with immunohistochemistry (IHC) in Lynch-related cancers from 2000 to 2020 and, more latterly, the impact of somatic next-generation sequencing (NGS) of tumours in individuals with protein loss on IHC in tumours but without a germline path_MMR.

## Methods

### Participants

Individuals referred to the regional genetics department in Manchester with an LS-related cancer and concerns about the possibility of LS provided consent for tumour and, if necessary, germline analysis. The great majority of evaluated patients had CRC or EC and were selected based on early age at diagnosis or fulfilling Bethesda guidelines or Amsterdam criteria. Occasional cases were tested as deceased first-degree relatives of clinically unaffected index patients. In addition, 500 women from the Proportion of Endometrial Tumours Associated with Lynch Syndrome (PETALS) study with sequential EC were also included (15/NW/0733).^25^ Generally, individuals fulfilling Amsterdam criteria did not undergo prescreening and went straight to germline path_MMR analysis.

The standard pathway for non-Amsterdam criteria tumours was an initial test for dMMR using IHC of the MMR proteins. If there was loss of MLH1, the samples were tested for *MLH1* promoter hypermethylation and the *BRAF* c.1799T>A (p.Val600Glu) pathogenic variant. Positive results for either of these are indicative of a somatic mutation of *MLH1*. All individuals with MLH1 loss, samples and wild type for *BRAF* and negative for promoter hypermethylation, as well as all sole PMS2 or MSH2/MSH6 loss, underwent germline lymphocyte testing where this was possible. *BRAF* c.1799T>A (p.Val600Glu) was suspended for EC once it was known this screen was not sensitive (0/23 tested were positive for c.1799T>A (p.Val600Glu).^26^

### Immunohistochemistry

IHC for the four MMR proteins was performed in the MFT clinical pathology laboratory using the automated Ventana BenchMark ULTRA IHC⁄ISH staining module and the OptiView, 3′diaminobenzidine V.5 detection system (Ventana Co, USA) according to standard clinical protocols.^25^ The proportion of stained tumour epithelial component/intensity of staining was scored by two expert independent observers using tumour stroma as internal control and as described elsewhere.^27 28^ Only tumours with complete loss of protein expression were reported as dMMR (not those with patchy loss).

### Methylation analysis

Reflex *MLH1* methylation testing was performed on tumours showing loss of MLH1 protein on IHC. Purified DNA was amplified with bisulfite-specific primers in triplicate. A region of the *MLH1* promoter containing four CpG dinucleotides whose methylation status is strongly correlated with *MLH1* expression was sequenced using a pyrosequencer (PSQ 96MA). Two independent scientists interpreted the pyrograms. ‘Hypermethylation’ described >10% mean methylation across the four CpG dinucleotides on two of three replicate analyses. A proportion of *MLH1* hypermethylation cases underwent reference standard germline MMR sequencing to exclude coexisting path_*MLH1* variants, usually when they had a significant family history. In addition to methylation, analysis testing was carried out for the *BRAF* c.1799T>A (p.Val600Glu) variant.

### Germline analysis

DNA was extracted from 2 to 5 mL lymphocyte blood (EDTA anticoagulant) using Chemagic DNA blood chemistry (CMG-1097-D) on an automated PerkinElmer Chemagic 360 Magnetic Separation Module and a JANUS Integrator four-tip Automated Liquid handling platform. DNA was eluted into 400 μL buffer. Extracted DNA samples were measured for DNA yield, concentration and quality using a Nanodrop ND-8000 spectrophotometer. MMR genes *MLH1*, *MSH2* and *MSH6* were amplified using long-range PCR followed by NGS using Illumina SBS V.2 2×150 bp and Illumina MiSeq to analyse the coding region, flanking sequences to ±15 bp and known splicing variants (minimum 100× coverage depth) of MLH1, MSH2 and MSH6.^25^ Variant identification and calling was via an in-house bioinformatic pipeline. Reported sequence changes and regions with <100× coverage were retested via Sanger sequencing using BigDye V.3.1. Copy number analysis to detect large genomic rearrangements affecting the MMR genes was performed using MLPA MRC-Holland probe mixes: P003-D1 *MLH1/MSH2* and P072-C1 *MSH6*. Variant nomenclature followed Human Genome Variation Society guidelines (http://www.hgvs.org/vamomen) using reference sequences: LRG_216, t1(*MLH1*); LRG_218, t1(MSH2); LRG_219, t1(*MSH6*). Exons were numbered consecutively starting from exon 1 as the first translated exon for each probe mix. Cases with PMS2 protein loss, normal MLH1 methylation and no path_*MLH1/MSH2/MSH6* variant underwent path_*PMS2* analysis at the regional specialist Yorkshire and North East Genomic Laboratory which included MLPA.

### Somatic tumour analysis

Tumour specimens were assessed by specialist pathologists. All tissues were formalin-fixed and paraffin-embedded according to local clinical protocols. Tissue blocks with the greatest tumour content (>70%) were chosen for DNA extraction. Tumour was either microdissected from 5×10 µm unstained sections or cored from tissue blocks, depending on tumour content. Non-malignant adjacent tissue was selected for comparative constitutional microsatellite instability (MSI) analysis. MMR genes *MLH1*, *MSH2* and *MSH6* were analysed as components of a somatic panel including *PTEN*, *TP53*, *APC*, *POLD1* and *POLE* using a custom NGS approach based on a Qiagen GeneRead amplicon based enrichment. *PMS2* was not assessed due to the difficulties with pseudogenes and high copy number variant rate. Formal loss of heterozygosity (LOH) analysis was not part of the initial panel but was introduced with microsatellite repeats after 12 months, but did not include LOH for *PMS2*.

### Statistics

Differences between values were tested by a two-tailed Fisher’s χ^2^ test.

## Results

**Figure 1:**
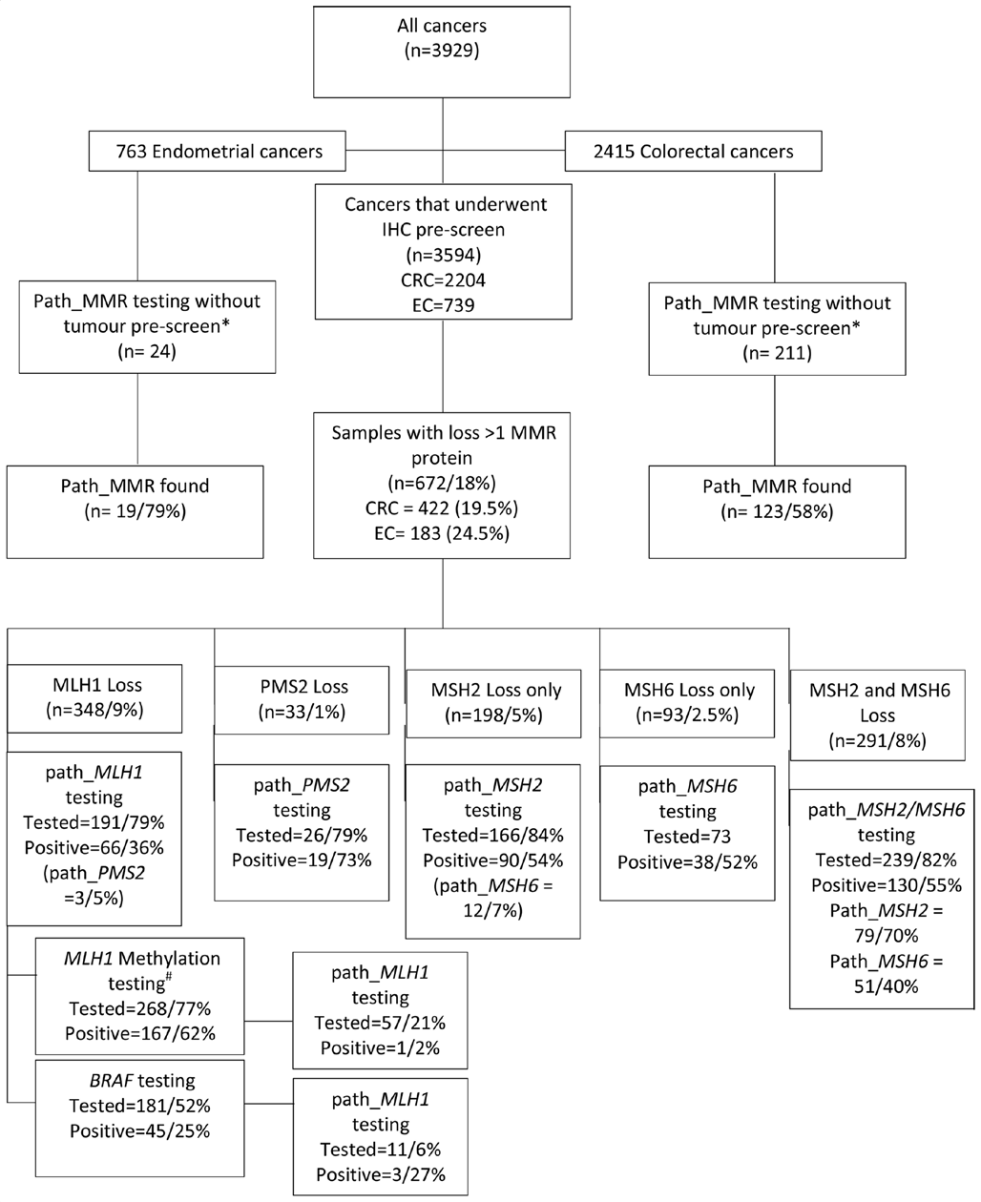
Study flowchart diagram. Note: germline testing was done for *MLH1*, *MSH2* and *MSH6* in cases and *PMS2* in select cases. Were a path_MMR was detected in a gene not consistent with the IHC loss, this is shown in brackets below the result. *The majority of these samples were Amsterdam criteria II positive. ^#^One sample had constitutional *MLH1* hypermethylation. EC, endometrial cancer; CRC, colorectal cancer; IHC, immunohistochemistry; MMR, mismatch repair; path_, pathogenic variant.

A total of 3694 index cases aged 8–91 years at diagnosis had a tumour (figure 1 and table 1) prescreened with tumour IHC of MMR proteins with 672 (18%) showing loss of at least one protein (table 2). CRC (n=2204 mean age 50.8 years) and EC (n=739 mean age=61 years) were by far the most frequently tested, and further analysis was largely confined to those two tumour types (online supplemental figure 1A, B). However, we also tested 761 other cancers and benign tumours (mean age=50, table 1).

10.1136/jmedgenet-2020-107542.supp1 Supplementary data

10.1136/jmedgenet-2020-107542.supp2 Supplementary data

**Table 1:** Tumour samples tested, age at diagnosis, IHC loss and path_MMR rate

	IHC (n)	Age range (median)	IHC loss	% IHC loss	Path_MMR with loss	%
Colorectal cancer	2204	14.5–91 (50.8)	422	19.15	155	7.03
Colorectal polyps	244	8.4–82 (54)	8	3.28	3	1.23
Endometrial cancer	739		183	24.76	44	5.95
Genetics service	239	16–79 (51)			28	11.7
PETALS	500	(65)			16	3.2
Gastric cancer	58	17–79 (48)	5	8.62	0	0.00
Ovarian cancer	261	16–89 (49)	27	10.34	8	3.07
TCC/kidney	13	32–61 (45)	3	23.08	1	7.69
Non-melanoma skin cancer	29	37–75 (57)	12	41.38	3	10.34
Cholangiocarcinoma	20	32–76 (50)	5	25.00	0	0.00
Pancreas	13	37–71 (53)	1	7.69	0	0.00
Brain	12	9–84 (48)	4	33.33	1	8.33
Breast	15	33–74 (49)	0	0.00	0	0.00
Small bowel including ampulla	21	29–72 (48)	1	4.76	0	0.00
Unknown primary	19	26–71 (40)	0	0.00	0	0.00
Oesophagus	16	21–61 (51)	1	6.25	0	0.00
Other	30		0	0.00	0	0.00
Total	3694		672		215	

TCC urinary tract; others include cervix (n=4), prostate (n=4), sarcoma (n=3), melanoma (n=3), thyroid (n=2) and lung (n=2).

IHC, immunohistochemistry; MMR, mismatch repair; path_, pathogenic variant; PETALS, Proportion of Endometrial Tumours Associated with Lynch Syndrome; TCC, transitional cell carcinoma.

**Table 2:** IHC loss and germline path_MMR detection rates in all index samples tested

All	Tested (n)	IHC loss	%	Tested germline (n)	Germline PV	% Germline	
MLH1 loss	3694	348	9.42	191	66*	34.55	*63 MLH1*, *3 PMS2*
PMS2 loss alone	3694	33	0.89	26	19	73.08	*19 PMS2*
MSH2 loss	3694	198	5.36	166	90	54.22	*79 MSH2, 11 MSH6*
MSH6 loss	3694	215	5.82	176	102	57.95	*51 MSH6*, *51 MSH2*
Either MSH2 or MSH6	3694	291	7.88	239	130	54.39	*79 MSH2*, *51 MSH6*
Any loss	3694	672	18.19	456	215	47.15	
MSH6 loss alone	3694	53	1.43	73	38	52.05	*38 MSH6*
	**Tested (n)**	**Positive (n)**					
MLH1 loss hypermethylation	268	167†	62.3	57	1	1.75	*MLH1*
MLH1 loss *BRAF* c.1799T>A	181	45	24.86	11	3	27.3	
No loss	3022	0	0	329	19	5.78	5 *MLH1*, *7 MSH2*, *3 MSH6*, *4 PMS2*

*This rose to 65/134 (48.5%) unmethylated samples.

†One patient with colorectal cancer had germline *MLH1* methylation.

EC, endometrial cancer; IHC, immunohistochemistry; PV, pathogenic variant.

A total of 211 patients with CRC underwent germline MMR testing without an IHC prescreen with 123 (58%) demonstrating a pathogenic variant (56 *MLH1*, 59 *MSH2* and 8 *MSH6*). Similarly, 24 women with EC fulfilling Amsterdam criteria went direct to germline testing, of whom 19 (79%) had a path_MMR (4 *MLH1*, 10 *MSH2*, 4 *MSH6* and 1 *PMS2*).

Of the 672 tumours with dMMR IHC loss in prescreened samples (table 2), loss of MLH1 was most common (9.4%) with 7.9% having loss of either MSH2 or MSH6 or both. There were 215 path_MMR present in 456 lymphocyte samples tested (47.4%–63 *MLH1*, *79 MSH2*, *51 MSH6* and *22 PMS2*). The relatively low detection rate of only 34.5% for those with MLH1 loss is partially explained by screening of 57 samples showing MLH1 methylation of which only one had a path_*MLH1* germline variant (26 samples from PETALS and 31 clinical samples were tested). The patient with a germline *MLH1* had a caecal tumour aged 45 and met Amsterdam criteria. Thus, the true rate of *MLH1* promoter methylation-negative samples in this group was 65/134 (48.5%). The highest detection rates of path_MMR were for those with PMS2 loss alone (73%) and MSH6 loss alone (58%). Overall 12/22 (54.5%) with a *PMS2* germline path_MMR had a large rearrangement.

Table 3 shows the dMMR tumours and the pathogenic variants detected for CRC and EC, respectively. Overall, 2204 index CRCs underwent IHC and 422 (19.1%) showed dMMR with the highest proportion demonstrating MLH1 loss (10%). For EC, 183/739 (24.8%) samples were dMMR with 15% demonstrating MLH1 loss. Both overall dMMR rates (p=0.001) and MLH1 loss rates (p=0.0003) were significantly higher in EC, although the difference is entirely driven by MLH1 loss. *MLH1* promoter hypermethylation rates were much higher in EC at 87% (95/109) compared with only 41% (67/163) in CRC (p<0.0001). *BRAF* c.1799T>A was identified in only 26.5% (45/170) dMMR CRC samples, compared with 41% (67/163) with *MLH1* promoter hypermethylation (p=0.005). *BRAF* testing was of no value in EC and was abandoned for the PETALS study.^25^ The difference in path_MMR variant rates between CRC and EC was most striking for *MSH6* with pathogenic variants identified in 24/44 (54.5%) cases of EC compared with 21/155 (13.5%) for CRC (p<0.0001). Equally, *MLH1* variants were more common in CRC with 61/155 (39%) pathogenic variants compared with 2/44 (0.5%) in EC (p<0.0001).

**Table 3:** IHC loss and germline path_MMR detection rates in CRC and EC index samples tested

Colorectal	Number tested	IHC loss	%	Number tested germline	Germline path_MMR	% Germline path_MMR	
MLH1 and PMS2 loss	2204	171	7.8	104	40‡	38.5%	*37 MLH1*, *3 PMS2*
MLH1 loss alone	2204	51	2.3	33	24‡	72.7%	*24 MLH1*
PMS2 loss alone	2204	25	1.1	21	14	66.7%	*14 PMS2*
MSH2 and MSH6 loss	2204	81	3.7	76	54	71.0%	*48 MSH2*, *6 MSH6*
MSH2 loss alone	2204	45	2.0	32	8	25.0%	*8 MSH2*
MSH6 loss alone	2204	41	1.9	30	15	50.0%	*15 MSH6*
Either MSH2 or MSH6	2204	175	7.9	140	77	55.0%	*56 MSH2*, *21MSH6*
Any loss	2204	422	19.1	298	155	52.0%	
	**Tested (n)**	**Positive (n)**					
MLH1 loss hypermethylation	163	67*	41.1	23	1	4.3%	*MLH1*
MLH1 loss *BRAF* c.1799T>A	170	45	26.5	11	3	27.3%	
No loss	1782	0	0.0	176	18†	10.2%	4 *MLH1*, *7 MSH2*, *3 MSH6*, *4 PMS2*
**Endometrial**C							
MLH1 and PMS2 loss	739	108	14.6%	43	2†	4.6%	*2 MLH1*
MLH1 loss alone	739	4	0.5	4	0	0%	
PMS2 loss alone	739	7	0.95	5	5	100.0%	*5 PMS2*
MSH2 and MSH6 loss	739	32	4.3	29	16	55.2%	*12 MSH2*, *6 MSH6*
MSH2 loss alone	739	2	0.3	2	1	50%	*1 MSH2*
MSH6 loss alone	739	30	4.1	30	18	60.0%	*18 MSH6*
Either MSH2 or MSH6	739	64	8.7	61	37	60.7%	*24 MSH6*, *13 MSH2*
Any loss	739	183	24.8	113	44	38.9%	
	**Tested (n)**	**Positive (n)**					
MLH1 loss hypermethylation	109	95	87.2	32	0	0.0%	
MLH1 loss BRAF c.1799T>A	Not systematically tested						
No loss	556	–	0.0	120	1	0.8%	1 *MSH6*

*One patient with CRC had germline *MLH1* methylation, #13/74 (17.6%) Amsterdam criteria, 5/102 (4.9%) non-Amsterdam.

†This rose to 2/15 unmethylated samples.

‡This rose to 63/114 (55.3%) of unmethylated samples.

CRC, colorectal cancer; EC, endometrial cancer; IHC, immunohistochemistry; MMR, mismatch repair; path_, pathogenic variant.

A subset of samples with EC and CRC still underwent full germline testing despite no IHC loss. This was in general because of a strong family history, and it remains routine practice to test all Amsterdam criteria cases regardless of IHC loss.^27^ For CRC, 13/74 (17.6%) Amsterdam criteria and 5/102 (4.9%) non-Amsterdam criteria cases tested positive for a path_MMR despite normal IHC. However, for EC, only 1/120 (0.83%) tested positive for a path_*MSH6* and nil in other MMR genes. The higher figures for CRC may be because these are more common, and a sporadic tumour without IHC loss could explain at least some of these false negative results. Sensitivity for IHC loss in CRC was 155/173 (89.6%) and that for EC was 44/45 (97.8%), although these figures might drop further if all samples with retained IHC staining were tested.

The results of somatic analysis are shown in table 4 for CRC and EC, respectively. In seven cases with IHC loss, the individuals were deceased and a germline path_MMR was found on somatic testing and confirmed in normal tissue material. Initial somatic MMR testing in the first 40 CRC samples did not include an analysis of LOH, and thus many variants were only monoallelic. Overall, 15/20 monoallelic CRC samples did not have a formal LOH analysis. However, in EC, 17/18 tumours showed at least one somatic variant with 13/18 (72%) biallelic. Four tumours had a single variant at low allele frequency (*MSH2* c.2458+1G>A (5.04%), *MSH2* c.1003dupA (9.5%), *MSH6* c.3261delC (10.1%) and *MSH6* c.718C>T (13.2%)), which precluded a sensitive LOH analysis. Of the 183/739 (24.7%) EC samples showing IHC loss, 95 (52%) were explained by *MLH1* promoter methylation; 44 (24%) had a germline path_MMR; and 17 (9.3%) showed evidence of somatic involvement of the relevant gene. This leaves only 27/183 (14.7%), but six samples did not undergo germline testing and three did not have promoter methylation with MLH1 loss. Most of the remainder, bar 1, did not have tumour somatic testing. Assuming the same detection rates for somatic testing as in the 18 EC samples, this would leave no more than 3 of 183 (1.6%) unexplained and only 3/739 (0.4%) of the whole IHC prescreened cohort. Of the 422/2204 (19.15%) CRC samples showing IHC loss, 67 (15.9%) were explained by MLH1 promotor methylation; 155 (36.7%) had a germline path_MMR; and 65 (15.4%) showed evidence of somatic involvement of the relevant gene. This leaves 135/422 (32%), but 73 samples did not undergo germline testing as patients were deceased and 17 did not have promoter methylation with MLH1 loss.

**Table 4:** NGS somatic analysis on CRC and EC with IHC loss

IHC loss	Number	Hypermethylation	Germline from tumour	Germline negative blood	Somatic	No cause found	Cause of IHC loss found
Colorectal somatic testing							
MLH1/PMS2	47	0/46	4 MLH1*	43	30 *MLH1*	13	34/47 (72%) 10/34 monoallelic
MSH2/MSH6	38	nt	4 2 MSH6 2 MSH2	34	27 8 *MSH6* 19 *MSH2*	7	31/38 (82%) 10/38 monoallelic
Endometrial somatic testing							
MLH1/PMS2	5	0/5	0	5	4 *MLH1*	1	4/5 (80%) 3/5 double somatic†
MSH2/MSH6	13	nt	0	13	7 *MSH6* *6 MSH2*	0	13/13 (100%) 10/13 double somatic

For CRC: 13 MLH1 loss no cause found 2/3 MSH−1 double somatic *PTEN, 1 POLD1*.

7 MSH2 loss no cause found 4/5 MSS? Overcall: 1 MSH double somatic *PTEN*.

*One mosaic low level 16% VAF missed on germline testing found after tumour somatic c.1975C>T p.(Arg659Ter) MLH1.

†Most samples with monoallelic variants had allele frequencies of <10%, which precludes LOH analysis.

CRC, colorectal cancer; EC, endometrial cancer; IHC, immunohistochemistry; LOH, loss of heterozygosity; NGS, next-generation sequencing; VAF, variant allele frequency.

## Discussion

We have reported IHC tumour prescreening in 3694 tumour samples, which, to our knowledge, is the largest such series in the literature. Although IHC does not have 100% sensitivity,^12–14^ it has the advantage of identifying the relevant likely genes involved and allows targeted *MLH1* promotor methylation in a lower number of samples than MSI testing. We have previously shown that for EC, in particular with the higher rates of involvement of *MSH6* (55% in the present study), MSI is significantly less sensitive (58%) with IHC detecting 100% of 16 path_MMR.^25^ The high proportion of *MSH6* in EC is confirmed in other studies with 5/9 (55.5%) in a US universal testing study.^29^ An Australian study limiting testing to ECs<60 found 10/22 (45.4%) of those with path_MMR had a path_*MSH6*.^30^ The study confirms the utility of *MLH1* promotor methylation particularly for EC with 87% of MLH1 loss being explained. This is similar to the 86.3% in a meta-analysis of 29 studies with 1159 showing loss of MLH1.^31^ Although *MLH1* promoter methylation is less useful in CRC, it is still superior to *BRAF* testing. The significantly higher rates of promoter methylation in EC seem to account entirely for the higher rates of MLH1 loss. As methylation is a mechanism that is used to coordinate menstruation in the endometrium,^32^ we propose that there is increased opportunity for regions in the DNA to be erroneously methylated, which may explain increased promoter methylation of MLH1 in EC.

The current study has confirmed the high predictive value of isolated loss of PMS2 and, to a lesser extent, MSH6,^33^ although isolated MLH1 loss was also quite specific with 73% being caused by a germline path_MMR. We have also shown the importance of somatic MMR testing in cases with IHC loss unexplained by either *MLH1* promotor methylation or a germline path_MMR. Somatic bilalleic path_MMRs are found in a high proportion of these cases. Of the 284 patients with non-methylated MMR loss in a joint Ohio and Icelandic cohort, 157 had a germline path_MMR, (55%) and 92 (32.4%) had probable biallelic (double) somatic variants.^33^ They concluded that 19 (6.7%) were unexplained and 17 had incorrect IHC. While we demonstrated this well in EC, it was less well shown in CRC. This may be due to low neoplastic cell counts that preclude a sensitive assessment of LOH. Recutting tumour FFPE sections for higher neoplastic content may well overcome this issue. Furthermore, some IHC loss may be spurious (an overcall) and reanalysis or assessment of MSI in those that still remains with unexplained IHC loss may resolve the issue. For EC we have shown that <1% of cases undergoing IHC are left with an unresolved diagnosis. In reality, ‘Lynch’ like syndrome, which was thought to be due primarily to missed path_MMR or another inherited mechanism, appears to be a relatively uncommon situation once somatic testing has been performed especially in EC.

Although there was a low rate of path_MMR in patients with tumours with MLH1 loss on IHC and promoter methylation as a prescreen, we have previously demonstrated that 4/71 (5.6%) individuals with CRC and germline pathogenic variants in *MLH1* had evidence of promoter methylation.^27^ Three of these four fulfilled Amsterdam criteria did not have an IHC prescreen (they were tested after path_MMR was found); therefore, overall *MLH1* promotor methylation still left a >10% chance of a germline path_MMR. Similarly, those with Amsterdam criteria who had proficient MMR tumour on IHC also had a path_MMR rate above 10%. As such, we would still recommend that those with CRC fulfilling Amsterdam criteria undergo germline path_MMR testing irrespective of the IHC or *MLH1* promoter methylation result. The same may not be true for EC with the much higher rates of *MLH1* promoter methylation and low rate of pathogenic germline variants in those tested with proficient MMR on IHC.

Our results show the value of a combined tumour somatic and germline test after IHC loss. This can especially be seen in the population-based PETALS study where the 3.2% detection rate for germline path_MMR in EC is similar to that seen in unselected CRC. By combining a tumour somatic approach with germline testing in 500 ECs, this comprehensive testing left just 1.9% (2/106) MMR deficient tumours unexplained by a path_MMR variant/epigenetic silencing.^25^ As such, only 2/500 (0.4%) were left still in the Lynch-like category after testing. A similar mainstreaming approach for CRC as well as EC would leave far fewer with an uncertain diagnosis, and only those with a path_MMR or unexplained IHC would need referral to genetics. Unfortunately, we cannot be certain the results would be as good in CRC based on our analysis as this did not involve LOH analysis for many samples, but others have found a high rate of double somatic events in CRC.^33^ While families can be reassured when double somatic events account for IHC loss this will still leave some where the age of the patient or family history requires ongoing management as Lynch-like. Testing of benign colorectal polyps is quite specific but does not have a high yield, although it can detect germline path_MMR in individuals with strong family histories suggestive of LS.

There are some limitations to the present study. We did not perform MSI testing on all the samples with IHC and cannot therefore make a direct comparison, although for EC, we have previously shown reduced sensitivity of MSI.^25^ The selection criteria for testing for CRC was stronger than for EC, and therefore comparisons are likely to overestimate the contribution of IHC loss in CRC compared with the EC tested in this study. Nonetheless, this is likely to strengthen further some of the differences identified between CRC and EC. We also did not typically prescreen individuals meeting Amsterdam criteria, meaning that the detection rates for IHC loss and path_MMR may be underestimated compared with studies that included individuals meeting Amsterdam criteria. We would still test patients meeting Amsterdam criteria even if they had hypermethylation of *MLH1* as evidenced by the case presented here. Some authors now advocate starting analysis with a tumour somatic approach.^33^ It is certainly plausible that this will become more mainstream and may reduce the requirement for a prescreen for LS testing. However, given the high rate of copy number variants in LS (11%–46%)^34^ and especially in this study for *PMS2* (54.5%), the sensitivity to detect these in tumour samples needs to be fully validated first. *PMS2* is known to be difficult to screen in lymphocyte DNA, and therefore testing in stored non-frozen tissue samples requires a bespoke approach.

In conclusion, we have undertaken prescreening of a very large series of tumour specimens with IHC for dMMR. Detection rates for germline path_MMR are similar to previous estimates. We have shown the superiority of *MLH1* promoter hypermethylation over *BRAF* testing and the higher utility in EC compared with CRC. Furthermore, we have shown that somatic MMR testing with NGS removes most patients from the ‘Lynch’-like category with previously unexplained IHC loss.

## Data Availability

Data are available upon reasonable request from the corresponding author.
