# Supplementary figures and images for: Advances in genetic technologies result in improved diagnosis of mismatch repair deficiency in colorectal and endometrial cancers
Source: J Med Genet. 2021 Jan 15;59(4):328–34. doi: 10.1136/jmedgenet-2020-107542 (PMC8961751; doi:10.1136/jmedgenet-2020-107542)

Supplementary figure 1a: CRC samples tested

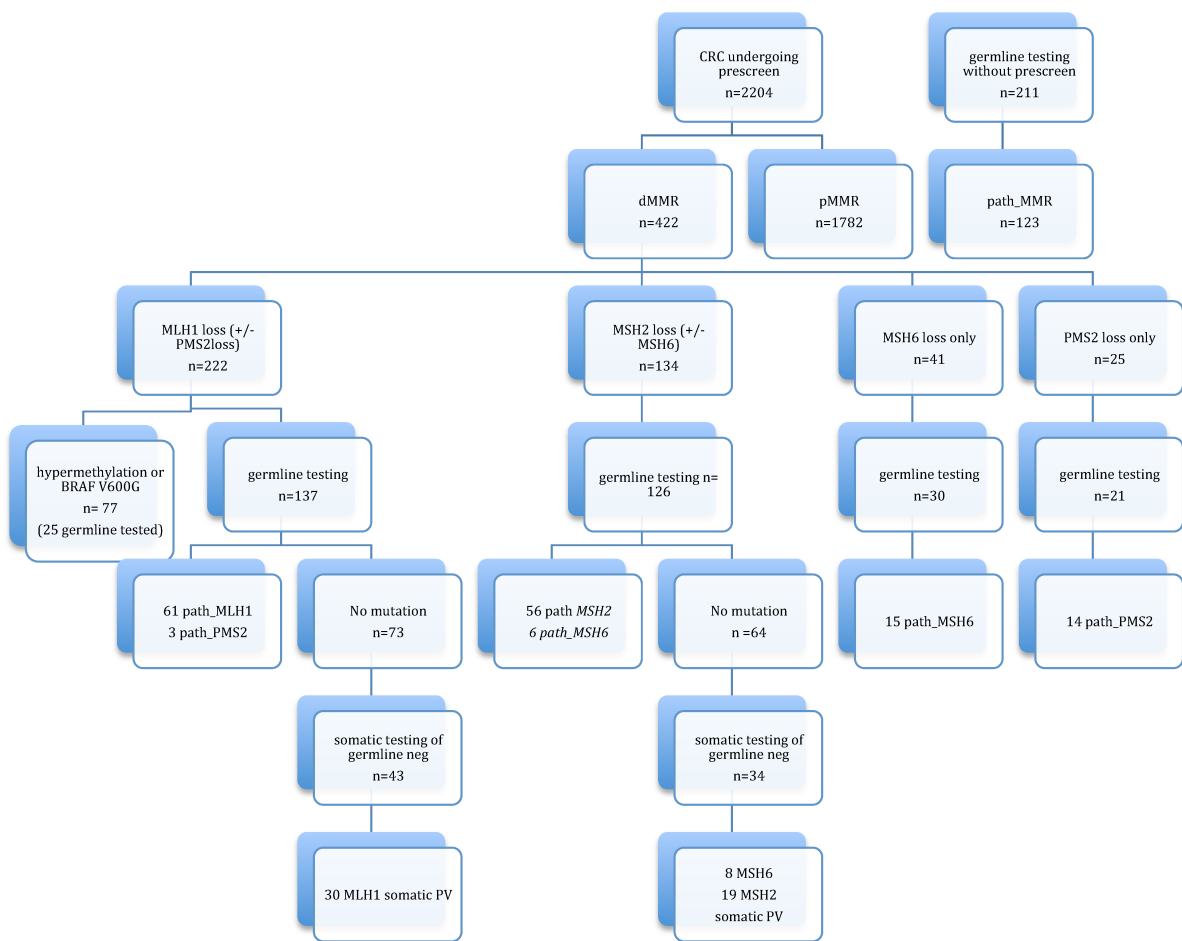

Supplement: Supplementary data [file jmedgenet-2020-107542supp001.pdf]

Supplementary figure 1b: EC samples tested.

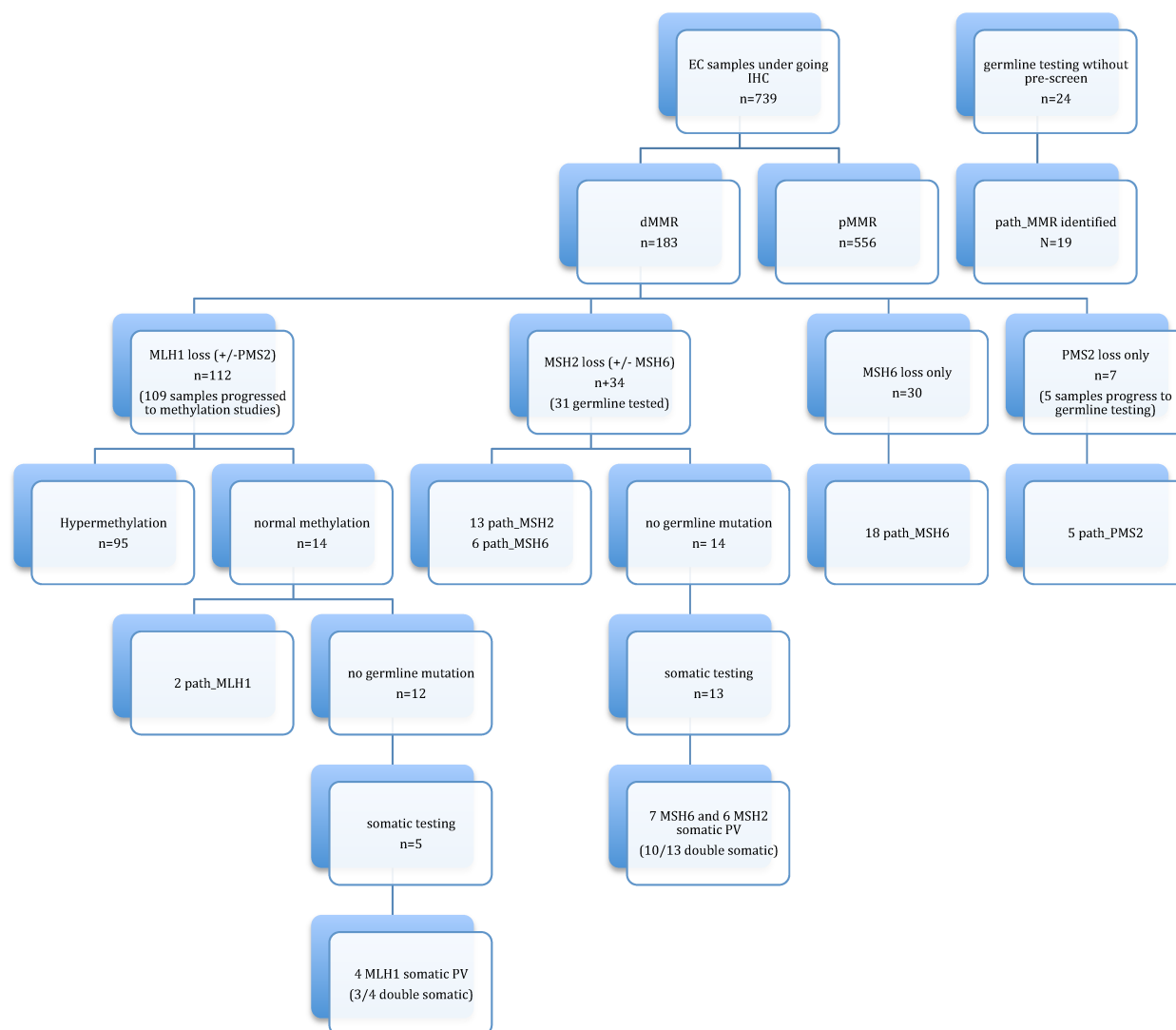

Supplement: Supplementary data [file jmedgenet-2020-107542supp002.pdf]
